# Diabetic Nephropathy: From Pathophysiology to Treatment

**DOI:** 10.1155/2017/2379432

**Published:** 2017-10-23

**Authors:** Ziyan Shen, Yi Fang, Tao Xing, Feng Wang

**Affiliations:** ^1^Department of Nephrology, Zhongshan Hospital, Fudan University, Shanghai, China; ^2^Shanghai Institute of Kidney and Dialysis, Shanghai, China; ^3^Shanghai Key Laboratory of Kidney and Blood Purification, Shanghai, China; ^4^Department of Nephrology, Shanghai Jiao Tong University Affiliated Sixth People's Hospital, Shanghai, China

Diabetic kidney disease (DKD) has been surging as the leading cause of end-stage renal disease (ESRD), as approximately one-half, in Europe, United States, Japan, and Taiwan. While in Mainland China, it has surpassed glomerulonephritis to become the number 1 cause of chronic kidney disease (CKD) in hospitalized population (1.10% versus 0.75%) in 2015 and with a substantially ascending rate doubling over the past one decade [1]. In clinical practice, some individuals with diabetes mellitus do not progress to DKD even if their blood glucose is not strictly controlled, or they are not so easily to progress from diabetic nephropathy (DN) into ESRD [2]. However, some individuals are inevitable to progress into ESRD with perfect blood glucose [3].

Thanks to international collaboration and novel analytical approaches, the underlying mechanism has been unraveled as a sophisticated model with interaction between hereditary basis and nonhereditary factors. The onset and progression of DKD are not only influenced by genetic profile but are also regulated by environmental, behavioral, and biological risk factors and their interaction with inherited predisposition. Several nonconventional risk factors may even play the critical role in the onset and progression of DKD.

The present special issue, which, including 9 original research articles and 4 review papers, has focused on the recent progress in our understanding of diabetic nephropathy including the underlying molecular mechanisms, genetic characteristics, new diagnostic biomarkers, and novel treatment options.

Studies about the genetic factors in DKD of T2DM are not so elucidated as in T1DM, since the concealing onset and the complicated phenotypes. Over the recent years, the penetration of candidate gene analysis by single-nucleotide polymorphisms (SNPs) and the more powerful genome-wide association studies (GWAS) allows dozens of genetic loci to be confirmed associated with DKD in T2DM. The efforts of screening out potential genes and SNPs are aiming to determine their role in the pathogenesis of DKD in T2DM. The localization of the gene on chromosome 18q22.3-23 was the first identified genetic loci in Turkish DKD patients of T2DM [4], which is in the region of carnosinase genes, and the polymorphism of relevant genes of carnosine dipeptidase (CNDP)1 and 2 was later proved to be related with the progression of DKD in T2DM patients [5, 6]. Two articles in this special issue targeted the genetic profiles of T2DM. Using genotyping techniques, L. Jin et al. found that the variant rs955333 was not associated with DKD, which was not consistent with the results of FIND, suggesting that the SNP might be less effective in eastern Chinese Han ancestry than other populations. In the work by T. Albrecht et al., the CNDP1 (CTG)5 homozygosity was identified as an independent, sex-specific protective factor for biopsy-proven DN. Their findings also suggested that hemodialysis patients with homozygous CNDP1 (CTG)5 genotype and diabetic patients carrying at least one (CTG)5 allele might have a survival benefit, yet to be confirmed by further studies.

Epigenetic modification also plays an important role in the pathogenesis of DN. A review by Z. Lu et al. presented recent advances in the epigenetics of DN, with the focus on the role of DNA methylation, noncoding RNAs, and histone modifications in DN [7–9].

Given that the diagnostic value of microalbuminuria in DN has recently been challenged by several studies, biomarkers other than urinary albumin are needed for the early identification of renal injury [10]. In this special issue, three clinical studies are focused on this profile. J. H. Kim et al. introduced brachial-ankle pulse wave velocity (baPWV) as a noninvasive marker associated with albuminuria in 2613 Korean patients with T2DM. A. Kamińska et al. reported that the density and size of urinary extracellular vesicles reflected renal function and could be considered as potential renal damage biomarkers in T2DM. N. Papadopoulou-Marketou et al. have conducted a small sample size cohort study to test the predictive role of serum neutrophil gelatinase-associated lipocalin (NGAL) on renal function and arterial blood pressure in T1DM. They found that serum NGAL served as an early biomarker of DN independently of microalbuminuria.

One review paper and two original research articles are related to the therapeutic options of DN. There are many ways to prevent or treat DN, but glycemic control is no doubt of paramount importance. In the clinical study titled “Renal Protective Effect of DPP-4 Inhibitors in Type 2 Diabetes Mellitus Patients: A Cohort Study,” Y-G. Kim et al. studied the efficacy of dipeptidyl-peptidase IV inhibitors (DPP-4i) in preventing DN among 414 T2DM patients and found that long-term treatment of DPP-4i could delay the progression of DN by reducing urine albumin excretion and alleviating eGFR decline. Animal models are competent tools to study novel therapeutic methods including DN. In the basic research work by Y. He et al., the authors established a rat model of islet transplantation under the kidney capsule and found that successful islet transplantation protected against DN better than insulin treatment, presenting a cheerful prospect for the treatment or prevention of early DN. The review titled “Mechanistic Insight and Management of Diabetic Nephropathy: Recent Progress and Future Perspective,” submitted by R. Xue et al., described recent advances about the pathophysiological process of DN and summarized emerging evidences for the management of DN.

Above all, the onset and progress of DKD is the consequence of genetic variants, epigenetic effects, and environment-involved interactions. Studies elucidating the underlying mechanisms behind DKD so far are rather limited, taking into account the paucity of well-phenotyped prospective DKD cohorts, the variable diagnostic criteria, the mosaic of ethnicity, and the cost-effective ratio of whole-genome genotyping. However, dozens of novel analysis technologies and pharmacological measures are booming out. To standardize the phenotypes, to larger the sample, to apply more stringent quality control, to replicate in multiple cohorts and to take covariates into account will no longer be a barrier in the near future. With the robust researches on the mechanisms within the onset and progression of DKD, the potential answers will ultimately be explored and will soundly shed light on the complex pathogenesis, facilitate prevention, and benefit early diagnosis and tailored intervention to reduce the incidence and minimize progression, so as to relieve the huge burden DKD posed on public health.
